# Evaluation of *Hydra* HALT-1 as a toxin moiety for recombinant immunotoxin

**DOI:** 10.1186/s12896-020-00628-9

**Published:** 2020-06-17

**Authors:** William F. Jiemy, Lih Fhung Hiew, Hong Xi Sha, Lionel L. A. In, Jung Shan Hwang

**Affiliations:** 1 Department of Biotechnology, Faculty of Applied Sciences, UCSI University, 56000 Kuala Lumpur, Malaysia; 2 Department of Biological Sciences, School of Science and Technology, Sunway University, No. 5, Jalan Universiti, Bandar Sunway, 47500 Selangor Darul Ehsan Malaysia; 3 Department of Medical Sciences, School of Healthcare and Medical Sciences, Sunway University, No. 5, Jalan Universiti, Bandar Sunway, 47500 Selangor Darul Ehsan Malaysia

**Keywords:** Actinoporin, Cnidaria, α-Pore forming toxin, Proinflammatory, Single chain fragment variable, Immunotoxin

## Abstract

**Background:**

Immunotoxin is a hybrid protein consisting of a toxin moiety that is linked to a targeting moiety for the purpose of specific elimination of target cells. Toxins used in traditional immunotoxins are practically difficult to be produced in large amount, have poor tissue penetration and a complex internalization process. We hypothesized that the smaller HALT-1, a cytolysin derived from *Hydra magnipapillata*, can be used as the toxin moiety in construction of a recombinant immunotoxin.

**Results:**

In this study, pro-inflammatory macrophage was selected as the target cell due to its major roles in numerous inflammatory and autoimmune disorders. We aimed to construct macrophage-targeted recombinant immunotoxins by combining HALT-1 with anti-CD64-scFv in two orientations, and to assess whether their cytotoxic activity and binding capability could be preserved upon molecular fusion. The recombinant immunotoxins, HALT-1-scFv and scFv-HALT-1, were successfully constructed and expressed in *Escherichia coli* (*E. coli*). Our data showed that HALT-1 still exhibited significant cytotoxicity against CD64^+^ and CD64^−^ cell lines upon fusion with anti-CD64 scFv, although it had half cytotoxic activity as compared to HALT-1 alone. As positioning HALT-1 at N- or C-terminus did not affect its potency, the two constructs demonstrated comparable cytotoxic activities with IC_50_ lower in CD64^+^ cell line than in CD64^−^ cell line. In contrast, the location of targeting moieties anti-CD64 scFv at C-terminal end was crucial in maintaining the scFv binding capability.

**Conclusions:**

HALT-1 could be fused with anti-CD64-scFv via a fsexible polypeptide linker. Upon the successful production of this recombinant HALT-1 scFv fusion protein, HALT-1 was proven effective for killing two human cell lines. Hence, this preliminary study strongly suggested that HALT-1 holds potential as the toxin moiety in therapeutic cell targeting.

## Background

Immunotoxin, often termed “targeted therapy”, is a hybrid protein consisting of a toxin moiety that is linked to a targeting moiety for the purpose of specific elimination of the target cells. The targeting moiety is generally a monoclonal antibody or genetically engineered antibody fragments. The first generation immunotoxins were created by chemically conjugating a monoclonal antibody with a toxin moiety. However, the first generation immunotoxins were large and therefore ineffective in tissue penetration and induced immunogenicity in the host [1]. The latest generation of recombinant immunotoxins were constructed by linking the gene encoding single-chain variable fragment (scFv) with the gene encoding the toxin moiety and expressing them in host cells. Although the latest generation of immunotoxins significantly reduce the molecular weight of the targeting moiety, the toxins commonly used are still too large. Most of these toxins such as RicinA, *Pseudomonas* exotoxin, and diphtheria toxin are 30–58 kDa and require internalization to the cytosol of target cells to work [1]. These properties lead to disadvantages such as low tissue penetration rate, defect in cytosol delivery and degradation of the immunotoxin in lysosomes before exerting their effect [2, 3]. A smaller sized toxin with no internalization process could eliminate these disadvantages.

HALT-1 (*Hydra* actinoporin-like toxin 1), a pore-forming toxin derived from *Hydra magnipapillata*, could be a new candidate for the toxin moiety in the recombinant immunotoxin [4]. HALT-1 resembles actinoporin, a family of α-pore forming toxins (α-PFTs) first identified in sea anemones but also found in other cnidarians [5]. Actinoporins such as equinatoxins and sticholysins bind to sphingomyelins on the cell membrane and create functional pores by oligomerization of four or more than four monomers, leading to an osmotic imbalance in the cell and subsequently cell lysis [6, 7]. Early attempts have been made to use equinatoxin II and sticholysin I & II as immunotoxins for anti-parasite and anti-cancer therapy [8–10]. One of these early immunotoxins was based on the disulphite linkage between the sticholysin and the monoclonal antibody IOR-T6 that bound directly to the antigen on the surface of immature T-lymphocytes [8]. This immunotoxin was highly toxic for IOR-T6 carrying cells (CEM) and not toxic for non-IOR-T6 cells (K562). Under reducing condition, sticholysin was released from immunotoxin and able to equally kill both cell types [8]. Another “prototype” used avidin and biotinylated secondary antibody to link two separate moieties, anti-*Giardia* antibody and biotinylated equinatoxin II, in the anti-*Giardia* assay [9]. The authors demonstrated quite promising results with respect to the specificity of the toxic effect of actinoporins on parasite cells. Although these actinoporin-based immunotoxins belong to the first or second generations of immunotoxin in which the targeting and toxin components are chemically conjugated in vitro, the actinoporins could exert cytolytic activity against targeted cells and were proven as good candidates for constructing immunotoxins. In recent studies, actinoporin is also known to cause cell death in a regulated manner. For example, intracellular ion imbalance that was due to the low-dose exposure of sticholysin II could activate the RIP1-MEK1/2-ERK1/2-pathways and subsequently induce the regulated necrosis-like cell death mechanism [11, 12]. Hence, actinoporins including HALT-1 are versatile proteins with multiple modes of action. Moreover, compared to other toxins used for the construction of immunotoxins, actinoporin or HALT-1 is much smaller in size (20.8 kDa) and works by forming pores on cell membrane, which may provide a solution to overcome the disadvantages of other toxins.

Macrophages have been identified as one of the major cellular players in the pathogenesis of numerous chronic inflammatory disorders including vasculitis [13], atherosclerosis [14], rheumatoid arthritis [15], systemic lupus erythematosus [16], making them an attractive target for immunotoxin development. A study by Thepen et al. [17] demonstrated a successful reduction of chronic cutaneous inflammation in a mice model by targeting inflammatory macrophages using CD64 targeted immunotoxin, H22-RicinA. Generally, macrophages are categorized into two distinct phenotypes, which are the M1 (classically activated, proinflammatory) and M2 (alternatively activated, tissue remodelling) macrophages [13, 15, 18]. It is important to note that the M1/M2 paradigm of macrophage polarization is an oversimplified classification based on in vitro model, which may not directly resemble macrophage behaviour in vivo. Nonetheless, strong activity of pro-inflammatory cytokines and reactive species, which clearly resembles the skew towards M1-like activation, has been associated with the development of persistent chronic inflammation [19]. Therefore, M1-like macrophages may serve as a potent therapeutic target for reducing chronic inflammation. Activated macrophages express a wide variety of cell surface markers. In general, these markers are expressed on both M1 and M2 macrophages. However, some markers are expressed in greater quantity on M1 macrophage and are downregulated on M2 macrophage. An example of such receptor is CD64 (Fc gamma receptor 1), a high-affinity immunoglobulin Fc receptor [20]. The high expression of CD64 on M1-like macrophages makes this receptor an attractive target for specific elimination of M1-like proinflammatory macrophages. Additionally, evidence has shown that CD64 is only expressed on myeloid cell lines including monocytes, macrophages and activated neutrophils [21, 22]. Numerous other studies have also shown reduction of inflammation with CD64 targeted immunotoxin further confirming the utility of CD64 as a target for immunotoxin development [23–25].

We hypothesized that HALT-1 could be used as a toxin moiety for the construction of recombinant immunotoxin. In this study, we described for the construction of HALT-1-based recombinant immunotoxins by molecular fusion of HALT-1 with anti-CD64 scFv in two different orientations. We then determined the binding potential of the two immunotoxins to CD64 in vitro. Moreover, the efficacy of these recombinant immunotoxins against M1-like macrophages and HeLa cells was evaluated in terms of the cytotoxicity of HALT-1, but not the selective binding affinity of scFv to cells expressing CD64 since the immunotoxins could recognise both M1-like macrophages and HeLa cells via HALT-1. Our findings suggested the potential of using HALT-1 as toxin moiety for construction of recombinant immunotoxins with preferable arrangement of HALT-1 at the N-terminal end. As the binding specificity of scFv to CD64 could have been masked by HALT-1 which recognises the membrane lipids of almost all human cell types, the immunotoxins did not differentiate CD64^+^ M1-like macrophages from CD64^−^ HeLa cells. Hence, the future study should replace HALT-1 with a mutant lacking the binding affinity to membrane lipids.

## Results

### In vitro assessment of α-CD64-scFv (or scFv) binding to CD64

**Fig. 1:**
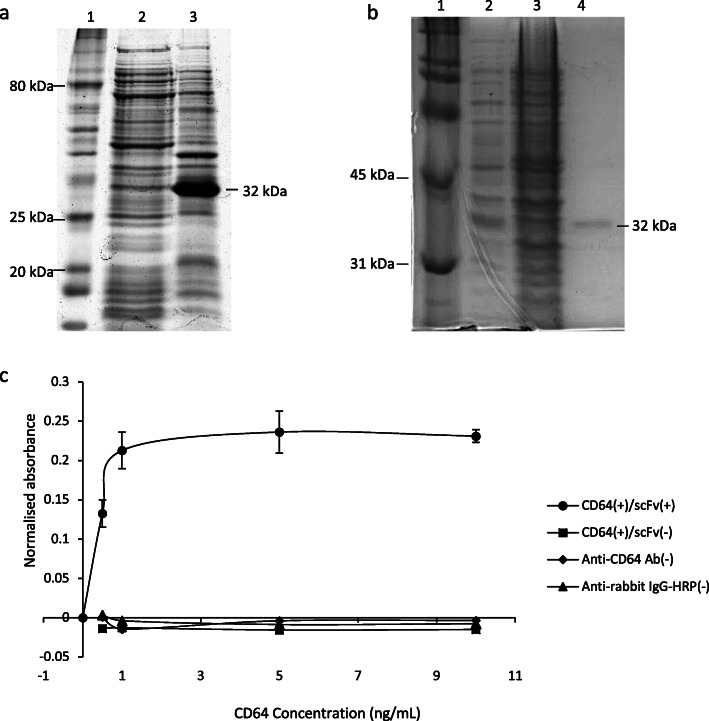
12% SDS-PAGE image and binding assay of α-CD64 scFv. **a** Expression of recombinant α-CD64-scFv. Lane 1, 10–250 kDa protein ladder; lane 2, soluble fraction; lane 3, insoluble fraction. The expected band of 32 kDa was observed in the insoluble fraction. **b** α-CD64-scFv after refolding in a series of deceasing urea concentrations. Lane 1, protein ladder, lane 2, *E. coli* cell lysate with the induction of IPTG; lane 3, *E. coli* cell lysate without IPTG, and lane 4, refolded α-CD64 scFv visible as the band of 32 kDa. **c** ELISA assay of α-CD64 scFv binding against various concentrations of CD64 (0, 0.5, 1.0, 5.0 and 10 ng/mL). Maximum binding (0.2362) was achieved at 5 ng/mL of CD64. Each point is the mean generated from triplicate reactions. The controls (no scFv, no anti-CD64 Ab, and no anti-rabbit IgG-HRP) showed no absorbance signal. See also Figure S1; Additional file 2

CD64 has been shown to be a good choice of target for the development of therapies against many kinds of monocyte/macrophages related inflammatory diseases such as rheumatoid arthritis, inflammatory skin diseases and acute myeloid leukemia (AML). α-CD64-scFv used in our study is based on the amino acid sequence of H22(scFv) that was reported to show specific binding towards CD64 [26–28]. α-CD64-scFv was expressed as a recombinant protein in BL21(DE3) *E. coli* cells and its solubility was assessed before the purification. Soluble lysate of expressed culture was compared with the insoluble cell debris on SDS-PAGE (Fig. 1a). Our results showed α-CD64-scFv present as an insoluble 32 kD protein. Hence it was purified under denaturing condition and then followed by refolding. The final yield of recombinant α-CD64-scFv was 144 μg/mL (Fig. 1b) with the recovery of slightly less than 40%.

The binding of α-CD64-scFv to CD64 at various concentrations was assessed by ELISA. In Fig. 1c, there was a sharp increase of absorbance from 0 to 1.0 ng/mL and then the absorbance remained constant even though the concentration of CD64 was increased to 10 ng/mL. This suggested that there was a direct proportional relationship between CD64 concentration and binding to α-CD64-scFv. As such, α-CD64-scFv is a potential vehicle for immunotoxin to be targeted to M1 macrophages and could be used in the production of immunotoxin.

### CD64-binding and cytotoxicity of HALT-1-scFv and scFv-HALT-1

**Fig. 2:**
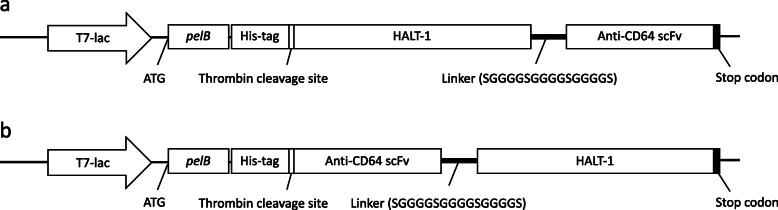
Fusion of HALT-1 with α-CD64-scFv. Schematic structure of the recombinant immunotoxins in pET22b expression vector. HALT-1 and α-CD64-scFv were connected via the peptide linker containing glycine and serine (SGGGGSGGGGSGGGGS). **a** N-terminal HALT-1 and C-terminal anti-CD64-scFv. **b** N-terminal anti-CD64-scFv and C-terminal HALT-1

Two recombinant immunotoxins with opposite orientations of α-CD64-scFv and HALT-1 were constructed, one with HALT-1 at the N-terminus and α-CD64-scFv at the C-terminus, and the other having HALT-1 at the C-terminus and α-CD64-scFv at the N-terminus. Figure 2 shows the schematic drawing of the recombinant immunotoxins in pET22b expression vector. Orientation of α-CD64-scFv and HALT-1 would determine whether or not the recombinant immunotoxin can be produced in *E. coli* and bind on the cell membrane to form the oligomeric pores. Many known immunotoxins have their own preference of moiety orientation. For instance, *Pseudomonas* exotoxin A is often positioned at the C-terminal end of immunotoxin [29, 30] whereas *Diphtheria* toxin tends to be placed at the N-terminal end [31, 32]. In this study, we prepared the recombinant immunotoxin in two different orientations in such that one might work better than the other.

**Fig. 3:**
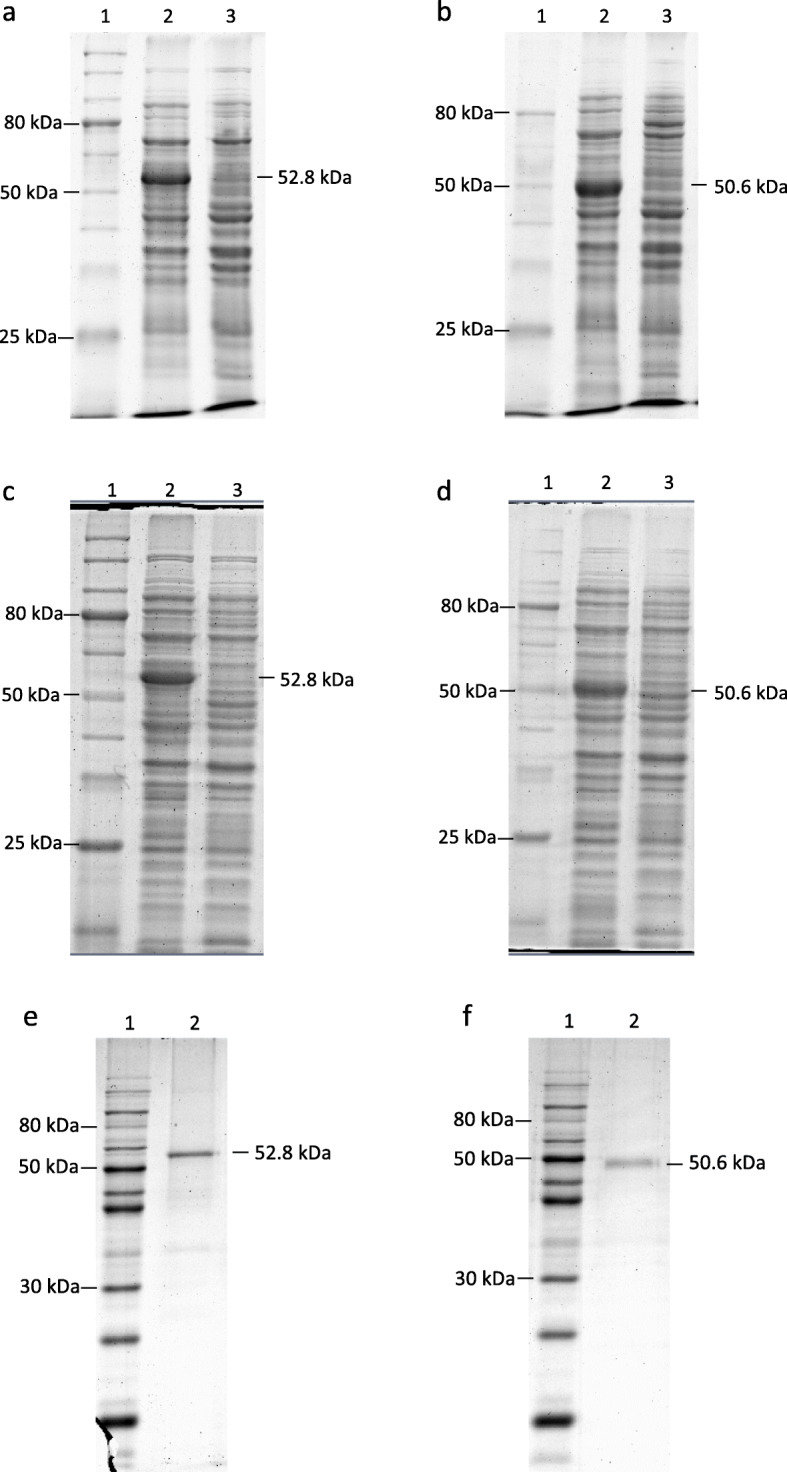
12% SDS-PAGE of the recombinant immunotoxins showing their expression, solubility and refolding yield. **a** Cell lysate was extracted after the expression of recombinant HALT-1-scFv in BL21(DE3) *E. coli* cells. Lane 1, 10–250 kDa protein ladder; lane 2, HALT-1-scFv in the presence of IPTG; lane 3, HALT-1-scFv in the absence of IPTG. **b** Cell lysate was extracted after the expression of recombinant scFv-HALT-1 in BL21(DE3) *E. coli* cells. Lane 1, 10–250 kDa protein ladder; lane 2, scFv-HALT-1 in the presence of IPTG; lane 3, scFv-HALT-1 in the absence of IPTG. **c** Solubility of HALT-1-scFv was examined after the cell disruption by sonication. Lane 1, 10–250 kDa protein ladder; lane 2, HALT-1-scFv insoluble faction; lane 3, HALT-1-scFv soluble fraction. **d** Solubility of scFv-HALT-1 was examined after the cell disruption by sonication. Lane 1, 10–250 kDa protein ladder; lane 2, scFv-HALT-1 insoluble faction; lane 3, scFv-HALT-1 soluble fraction. **e** Recombinant HALT-1-scFv after the refolding process. Lane 1, 12–120 kDa protein ladder; lane 2, HALT-1-scFv. **f** Recombinant scFv-HALT-1 after the refolding process. Lane 1, 12–120 kDa protein ladder; lane 2, scFv-HALT-1. See also Figure S2; Additional file 2

Both recombinant immunotoxins were successfully expressed in BL21(DE3) *E. coli* cells in the presence of 1 mM IPTG (Fig. 3a and b). However, both recombinant immunotoxins were present in insoluble inclusion bodies (Fig. 3c and d). The insoluble inclusion bodies were isolated, denatured, and purified with Ni-NTA column before the refolding procedure. Stepwise dialysis refolding was processed over a long period of time to achieve high refolding efficiency and recovery of bioactive immunotoxin. Then we confirmed their purity by SDS-PAGE (Fig. 3e and f). The total yield after refolding was below 40%, indicating that less than 40% of recombinant immunotoxins were refolded properly, soluble and biologically active (Supplementary Table 3). Those that left unfolded or misfolded would remain as aggregates.

**Fig. 4:**
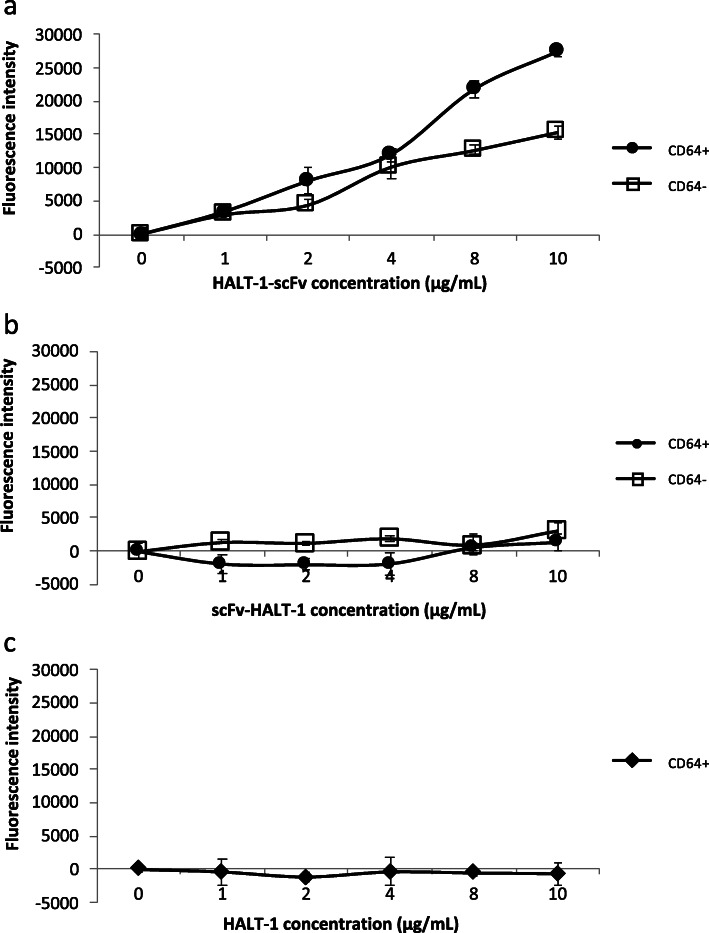
ELISA assay of CD64 binding to recombinant immunotoxins. **a** HALT-1-scFv; **b** scFv-HALT-1; **c** HALT-1. Various concentrations of HALT-1-scFv, scFv-HALT-1 and HALT-1 (0, 1, 2, 4, 8 and 10 μg/mL) were used in the assay with or without CD64. Each assay was performed in triplicate and error bars represent standard deviations from the mean of triplicate. Recombinant HALT-1 alone was used as a control to indicate that non-specific binding did not occur between HALT-1 and CD64. Fluorescence intensity was obtained by subtracting the fluorescence value with 0 μg/mL of either HALT-1-scFv, scFv-HALT-1 or HALT-1

To evaluate whether α-CD64-scFv is still able to bind CD64 after it has coupled with HALT-1 in the immunotoxin, we did an ELISA assay. A CD64-coated 96-well immunoplate was treated with various concentrations of the recombinant immunotoxins, HALT-1-scFv and scFv-HALT-1. Our results showed concentration-dependent binding of recombinant HALT-1-scFv to CD64 from 0 to 10 μg/mL (Fig. 4a). Although HALT-1-scFv also showed weaker non-specific binding towards 3% BSA in CD64(−) control wells, its binding to CD64 was significantly one-fold higher as compared with CD64(−) control (One Way Repeated Measures ANOVA *p* = 0.0381) (Fig. 4a). scFv-HALT-1, on the other hand, showed no binding to CD64 (Fig. 4b). This might be due to the incorrect folding of α-CD64-scFv during the refolding step and subsequent loss of its binding activity towards CD64. Figure 4c demonstrated that the HALT-1 moiety did not contribute to the CD64 binding of the recombinant immunotoxins.

**Fig. 5:**
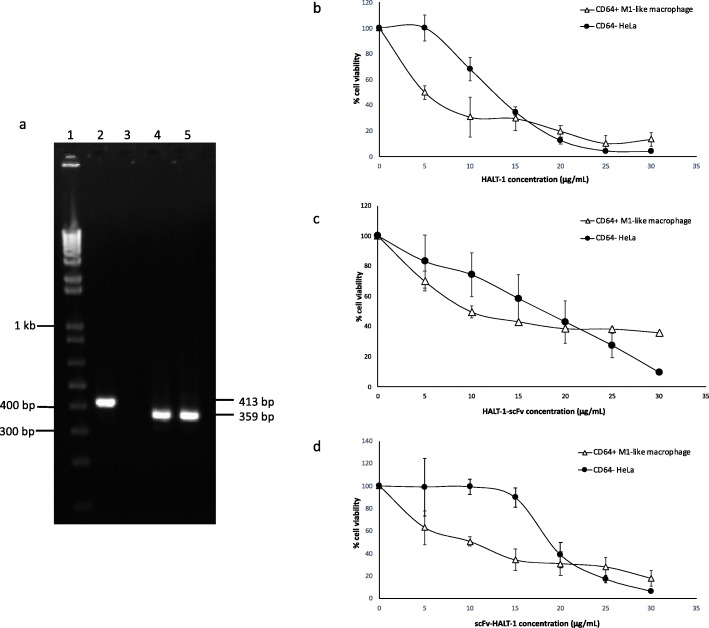
Cytotoxicity of recombinant immunotoxins towards CD64^+^ M1-like macrophages and CD64^−^ HeLa cells. **a** PCR validation of CD64 expression. Lane 1, 1 kb plus DNA ladder; lane 2, CD64 expression in M1-like macrophage; lane 3, CD64 expression in HeLa cells; lane 4, GAPDH expression in M1-like macrophage; lane 5, GAPDH expression in HeLa cells. **b**, **c**, **d** Cytotoxicity of HALT-1 alone and recombinant immunotoxins was measured at increasing concentrations (0, 5, 10, 15, 20, 25 and 30 μg/mL) against CD64^+^ M1-like macrophages and CD64^−^ HeLa cells. **b** HALT-1; **c** HALT-1-scFv; **d** scFv-HALT-1. Data are presented as mean ± standard deviations from triplicate experiments. See also Figure S3; Additional file 2

Cytotoxicity of the recombinant immunotoxins were assessed in vitro by measuring viability of CD64^+^ M1-like macrophages and CD64^−^ HeLa cells treated with various concentrations of immunotoxins. Before proceeding with the cytotoxicity assay, PCR was utilized to validate the expression of CD64 in activated M1-like macrophages as well as the lack of CD64 expression in CD64^−^ HeLa cells. The results clearly demonstrated the expression of CD64 in M1-like macrophages and the lack of CD64 expression in HeLa cells (Fig. 5a). Figure 5b, c and d display the viability of CD64^+^ M1-like macrophages and CD64^−^ HeLa cells following treatment with increasing concentrations of HALT-1, HALT-1-scFv and scFv-HALT-1. CD64^+^ M1-like macrophages are slightly more susceptible than CD64^−^ HeLa cells to the cytotoxicity of HALT-1, either alone or in conjugation with scFv (Fig. 5b, c and d). HALT-1 toxin alone has an IC_50_ of 5.05 μg/mL against CD64^+^ M1-like macrophages and 12.55 μg/mL against CD64^−^ HeLa cells (Fig. 5b). HALT-1-scFv was shown to have cytolytic activity with IC_50_ of approximately 10.05 μg/mL on CD64^+^ M1-like macrophages while its IC_50_ towards CD64^−^ HeLa cells was found at 17.95 μg/mL (Fig. 5c). Despite the absence of CD64 on the cell membrane of CD64^−^ HeLa cells, HALT-1-scFv still exerted cytotoxicity as the HALT-1 moiety could recognise its target sites on the cell membrane. Both CD64^+^ and CD64^−^ cells were also killed by scFv-HALT-1 at IC_50_ of 9.95 μg/mL and 18.8 μg/mL, respectively (Fig. 5d). Since scFv-HALT-1 immunotoxin did not bind CD64 (Fig. 4b), this cytotoxicity must be due to the activity of the HALT-1 moiety. Therefore, the immunotoxins that we constructed in this study reduced the cell viability of CD64^+^ and CD64^−^ cells, but whether they selectively target CD64^+^ cells would require more study using mutant HALT-1 lacking receptor-binding activity.

Taken together these results showed that there were only small differences in the cell viability between cells treated with the two immunotoxins and HALT-1. This supports two conclusions: (1) the toxicity of HALT-1 was not significantly altered by the presence of scFv moiety in the recombinant immunotoxin and (2) the toxicity of immunotoxins was due primarily to HALT-1 binding to cells and was independent of the presence (M1-like macrophage) or absence (HeLa cells) of CD64 on the cell membrane.

## Discussion

Recombinant immunotoxins were successfully constructed by molecular fusion of HALT-1 and α-CD64-scFv via a flexible linker (Fig. 2). The serine and glycine-rich linker was selected due to several reasons; (1) flexible glycine-rich regions have been observed as natural linkers in multidomain proteins; (2) glycine and serine provide good flexibility due to their small sizes; (3) serine and glycine help maintain stability of the linker structure in the aqueous solvent by forming hydrogen bonds with water; (4) linker length is within the optimal length of 6 or 10 ± 5.8 residues [33, 34]. As stated above, pET22b expression vector was chosen because it contains pelB leader sequence to bring the protein to periplasmic space for disulfide bonds formation [35]. Both recombinant immunotoxins were expected to have the molecular weight of 50.6 kDa. However, the SDS-PAGE results indicated that the molecular weights of HALT-1-scFv was larger than expected (approximately 52.8 kDa, Fig. 3a) while the molecular weight of scFv-HALT-1 had the approximately correct size of 50.6 kDa (Fig. 3b). The increase in molecular weight of HALT-1-scFv is likely caused by the failure of the pelB leader sequence (approximately 2.2 kDa) to be cleaved off during expression. This result was corroborated with the subsequent solubility test (Fig. 3c) showing these immunotoxins were expressed as insoluble inclusion bodies which reflected the failure of periplasmic translocation and cleavage of pelB leader sequence. However, despite the successful cleavage of pelB leader sequence, scFv-HALT-1 was also present in the inclusion body (Fig. 3d). In future studies, eukaryotic expression system such as green alga could be used to improve the production and solubility of the recombinant immunotoxins [36].

Refolded recombinant HALT-1-scFv, but not scFv-HALT-1, showed in vitro binding to CD64. This suggested that HALT-1-scFv where HALT-1 and α-CD64-scFv were placed at the N-terminus and C-terminus, respectively, could be selected for further development of immunotoxin. However, HALT-1-scFv also showed significant degree of non-specific binding towards BSA. Reports have shown that different antibodies and proteins may exert cross-reactivity with BSA [37, 38]. The non-specific cross-reactivity towards BSA in our results might be one of the rare examples. Additionally, previous report has also shown that glycerol may differentially affect antibody-antigen interaction depending on the antibody clone and antigen [39]. The high concentration of glycerol used in our desalting buffer during the protein refolding may contribute to the signal in CD64(−) ELISA. Expression system that eliminates the need to refold the recombinant immunotoxin or an improved redox refolding system could eliminate the need of glycerol in the refolding protocol.

Our study aims to demonstrate whether HALT-1 could maintain its cytotoxic function after it was conjugated to the scFv if the conjugated scFv preserves its target binding capability, and what orientation of HALT-1 in recombinant immunotoxin that gives the highest efficacy in killing the target cells. A number of recombinant immunotoxins targeting CD64 have been developed and reported in several studies as listed in Table 1. Compared to H22(scFv)-ETA, H22(scFv)2-ETA, granzymeB-H22(scFv), and H22(scFv)-MAP, the IC_50_ of HALT-1-scFv was significantly higher at approximately 189 nM (equivalent to 10.05 μg/mL). This significantly higher working concentration can be explained by the requirement of four or more monomers of HALT-1 to oligomerize in close proximity to form a functional pore and induce cell lysis. Albeit the higher working concentration, HALT-1 based recombinant immunotoxin can be beneficial in selective toxicity. Since at least four monomers of HALT-1 are needed to oligomerize in close proximity, the recombinant immunotoxin could presumably have low cytolytic activity when cells have low and sparse expression of the target surface receptor. As such, HALT-1 based immunotoxin targeting CD64 can selectively eliminate M1-like macrophage that express high amount of the surface receptor. When comparing the cytotoxic activity of HALT-1 before and after conjugating with α-CD64-scFv, either HALT-1-scFv or scFv-HALT-1 displayed a two times lower cytotoxic activity than that observed for HALT-1 (Fig. 5). This is not surprising because the reduction of activity was also observed in GFP conjugated equinatoxin II (GFP-EqtII). The fusion of GFP to the C-terminus of equinatoxin II caused the toxin becoming less haemolytic than the native toxin [41]. Despite the opposite orientations, HALT-1 in HALT-1-scFv and scFv-HALT-1 demonstrated similar cytotoxic activity in either CD64^+^ M1-like macrophages (IC_50_ = 10.05 and 9.95 μg/mL, respectively) or CD64^−^ HeLa cells (IC_50_ = 17.95 and 18.80 μg/mL, respectively). One may expect scFv-HALT-1 to have much less activity than HALT-1-scFv since the N-terminus of HALT-1 is functionally important. The role of N-terminal α-helix of actinoporin has been previously reported for equinatoxin II. Gutierréz-Aguirre et al. [42] has shown that equinatoxin II mutant having substitution of valine at position 22 to tryptophan (V22W) could establish interactions with the interface of membrane and prevent it from insertion into the lipid bilayer. Similarly, the N-terminal α-helix of double cysteine mutant 8–69 (V8C and K69C) of equinatoxin II was immobilised in the oxidised form but regained lipid penetration in the reduced form [43]. The initial binding of mutant 8–69 on lipid membrane was not affected under the oxidative condition, indicating that the N-terminal α-helix is only involved in the second stage of membrane insertion [43]. Thus, the addition of bulky scFv to the N-terminus of HALT-1 might hinder the detachment of N-terminal helix from the core protein and subsequently its insertion into the lipid bilayer. However, the cytolytic activity of scFv-HALT-1 did not seem to be affected by the fusion of scFv. Having said that, scFv-HALT-1 may not be the choice of immunotoxin because it failed to bind CD64 in vitro in this study. Furthermore, HALT-1 exerted different cytotoxic activities against different human cell lines regardless whether it was connected to a single-chain scFv or not [44]. In our case, HALT-1 worked more effectively in M1-like macrophages than in HeLa cells (Fig. 5). In the next course of development, we will introduce mutation(s) in the receptor binding domain of HALT-1 so that the immunotoxin would be directed by its targeting moiety to CD64 expressing cells. Two residues of HALT-1, tryptophan at position 113 and tyrosine at position 129, have been previously substituted into alanine respectively and these mutants, which did not bind membrane lipid, are appropriate candidates as the toxin component of immunotoxin [45]. Moreover, flow cytometry can be introduced to evaluate the binding of HALT-1-scFv or scFv-HALT-1 to CD64 expressing cells in comparison with the commercial anti-CD64 antibody. Lastly, that efficacy of HALT-1 based recombinant immunotoxin can be improved by modification of the scFv moiety to bivalent tandem scFv. Bivalent tandem H22(scFv)2-ETA showed ten folds reduction in IC_50_ compared to H22(scFv)-ETA [25].

**Table 1:** IC_50_ of CD64 targeted recombinant immunotoxins

Recombinant immunotoxin	Cell line	IC_50_ (nM)	References
H22(scFv)-ETA	HL60	0.17	[26]
H22(scFv)-ETA	U937	0.14	[25]
H22(scFv)2-ETA	U937	0.014	[25]
granzymeB-H22(scFv)	U937	1.7–17	[40]
H22(scFv)-MAP	HL60	0.04	[27]
HALT-1-scFv	THP-1	189	This study
HALT-1-scFv	HeLa	339.96	This study
scFv-HALT-1	THP-1	196.64	This study
scFv-HALT-1	HeLa	371.54	This study

Almost all toxins currently used in developing recombinant immunotoxins require internalization to cytosol to exert their toxicity, which could lead to degradation of the immunotoxin in lysosome. By comparison, HALT-1 works on the cell surface without the needs for internalization into the cytosol, which avoids the complexity of entry mechanism and the degradation in lysosome. It has been argued that the necrotic action of HALT-1 could cause intracellular components to induce an inflammatory response in the neighbouring cells [46]. Recently, it became clear that HALT-1 at IC_50_ (0.51 μM or 10.61 μg/mL) could also induce an apoptotic pathway in HeLa cells and that the same apoptotic effect could be induced in other cell lines with similar IC_50_ values [44]. Hence, apoptosis could occur in HALT-1 treated cells when sub-lytic concentration of HALT-1 was used [44]. Interestingly, the apoptotic pathway induced by HALT-1 might not require the internalization of toxin. Recent studies of sticholysin II, a member of actinoporin family as mentioned above, have proven that the pore formation, if not for cell lysis, would lead to the ion efflux which subsequently activates the apoptotic signalling pathway [11, 12]. This feature differs HALT-1 from other bacterial α-PFT and thus HALT-1 can be an alternative candidate for the construction of immunotoxins.

## Conclusions

We fused HALT-1 to α-CD64-scFv via a flexible linker peptide and demonstrated that HALT-1 could be utilized as a toxin moiety in recombinant immunotoxins. Our preliminary data suggested that the positioning of α-CD64-scFv in immunotoxin is crucial for its binding to CD64 and HALT-1 has reduced half of its cytotoxicity following the conjugation with α-CD64-scFv. Despite the limitations, the small molecular size of HALT-1 and the ability to exert its toxicity without the need to be internalized, HALT-1 could be advantages compared to the toxins commonly used in the construction of recombinant immunotoxins.

## Methods

### Recombinant α-CD64 scFv

Humanised anti-CD64-scFv sequence was obtained from Genbank with the accession number AY585869. To synthesize α-CD64-scFv, its sequence was optimized using IDT DNA’s codon optimization tools for optimum expression in *E. coli*. The gene was synthesized by IDT DNA (USA) and then placed in pIDT-sMART vector. In order to express the recombinant scFv, the gene was digested with *Nhe*I and *Nde*I and subcloned into pET28a. Finally the cloned plasmid was transformed into BL21(DE3) *E. coli* cells.

### Recombinant HALT-1

Our group had previously constructed recombinant HALT-1 in pET28a and successfully expressed it in BL21(DE3) *E. coli* cells and purified it with the Ni^2+^ affinity chromatography [45].

### Construction of recombinant immunotoxins

Fusion of HALT-1 to α-CD64-scFv as well as introducing the glycine-serine peptide linker (SGGGGSGGGGSGGGGS) were performed by overlap extension polymerase chain reaction (OE-PCR) and could result in two different orientations, HALT-1 at N-terminus followed by α-CD64-scFv or vice versa (Fig.2a and b). Basic steps to achieve the different oriented fusion proteins are the same. Firstly, extension PCR was performed on HALT-1 and α-CD64-scFv using *Pfu* DNA polymerase (Nex-Bio, Malaysia) and two sets of primers for each orientation were prepared to introduce N-terminal His-tag, restriction enzyme sites and linker sequence (Supplementary Table 1). In brief, PCR started at 95 °C for 5 min, one cycle of 95 °C for 30 s, 50 °C for 30 s, 72 °C for 1 min and 30 s; and repeated for 35 cycles at 95 °C for 30 s, 58 °C for 30 s and 72 °C for 1 min and 30 s; and finally ended with 72 °C for 5 min. After extension PCR was performed, PCR products were fused in assembly PCR. Assembly PCR started at 95 °C for 5 min repeated for 15 cycles at 95 °C for 1 min, 60 °C for 45 s and 72 °C for 1 min and 30 s; and finally ended with 72 °C for 5 min. After completion of assembly PCR, respective forward and reverse primers were added to the reaction tube and amplification PCR was performed immediately. Briefly, amplification PCR started at 95 °C for 5 min repeated for 35 cycles at 95 °C for 1 min, 58 °C for 30 s and 72 °C for 2 min and 30 s; and finally ended with 72 °C for 5 min. PCR product was run in 1% agarose gel and desired bands were isolated, followed by purification with Wizard SV gel and PCR clean up system (Promega, USA). After the digestion of *Nco*I and *Xho*I, it was cloned into pET22b expression vector and transformed into BL21(DE3) *E. coli* cells. Plasmid DNA was purified using DNA-spin plasmid purification kit (iNtRON, Korea) and subjected to DNA sequencing for confirmation. In this study, we collectively called both α-CD64-scFv-HALT-1 (or scFv-HALT-1) and HALT-1-α-CD64-scFv (or HALT-1-scFv) as the recombinant immunotoxins.

### Expression and purification of recombinant α-CD64-scFv and recombinant immunotoxins

The expression and purification of recombinant α-CD64-scFv and recombinant immunotoxins were carried out separately. In general, recombinant protein was expressed in BL21(DE3) *E. coli* cells in the presence of 1 mM IPTG at 37 °C for 3 h. Expressed culture was then re-suspended in Tris-Cl buffer (20 mM; pH 8) containing 1 mM PMSF for sonication. Sonication was performed for a total of 10 min per sample on ice at 130 watts and 20 kHz. Both recombinant anti-CD64-scFv and immunotoxins were present in insoluble fraction. In brief, the insoluble fraction was collected and washed twice for 30 min each with inclusion body washing buffer (2 M urea; 20 mM Tris-Cl; 0.5 M NaCl; 2% Triton X-100; pH 8) followed by final washing with ice-cold Tris-Cl (20 mM; pH 8). Washed inclusion bodies were solubilized in solubilization buffer (8 M urea; 20 mM sodium phosphate; 50 mM 2-mercaptoethanol; pH 7.8) overnight at room temperature. Solubilized inclusion bodies were purified using Nickel NTA (Ni-NTA) resin (Qiagen, Germany) by pH gradient. Briefly, solubilized inclusion bodies were bound to the resin followed by washing and elution with purification buffer (8 M urea; 20 mM sodium phosphate; 0.5 M NaCl; 20 mM 2-mercaptoethanol) with reducing pH (pH 7.8, pH 6, pH 5.5 and pH 4.5). Elution fractions were subjected to SDS-PAGE electrophoresis and fractions with purified protein were pooled together for refolding.

### Protein refolding for recombinant α-CD64-scFv and immunotoxins

Recombinant protein purified in denaturing condition was refolded by stepwise dialysis in sodium phosphate buffer containing 0.5 M NaCl, 0.1 M (for 6 and 4 M of urea) or 0.5 M (for 2 and 1 M of urea) L-arginine and 50 mM 2-mercaptoethanol with decreasing concentration of urea (6, 4, 2 and 1 M) (Supplementary Table 2). Dialysis was performed with gentle stirring on ice for 3 h each buffer change until 1 M urea buffer which was done overnight followed by three changes of 1X PBS containing 30% glycerol for 3 h each. Without the addition of glycerol, 100% of the recombinant immunotoxins aggregated in the final desalting (data not shown).

### ELISA binding assays

To show α-CD64-scFv is specific for recombinant human CD64 (Sino Biological, USA), their interaction was demonstrated by the enzyme-linked immunosorbent assay (ELISA). In a 96-well microplate, wells were coated with 10 μg/mL of α-CD64-scFv overnight at 4 °C. The unoccupied protein-binding site was then blocked by 3% (w/v) BSA/PBS and further incubation at room temperature for 2 h. After rinsing the wells, various concentrations of recombinant CD64 (0, 0.5, 1.0, 5.0 and 10 ng/mL) were added to the wells and incubated at room temperature for 2 h. To visualize the α-CD64-scFv and CD64 interaction, rabbit anti-CD64 polyclonal antibody (Invitrogen, USA) was added to each well, and followed by anti-rabbit HRP complex (1: 5000) and TMB substrate complex (Thermo-Fisher, USA). The signal produced was read at 370 nm by multi-mode microplate reader (TECAN, Switzerland). Four negative controls with each lacking a specific component of ELISA, either α-CD64-scFv, CD64 protein, rabbit α-CD64 polyclonal antibody or α-rabbit HRP conjugate, were prepared for each 96-well microtiter plate.

ELISA was also performed to determine binding of recombinant immunotoxins to CD64. The basic procedure was carried out as described above. Recombinant human CD64 (0.5 μg/mL) was first coated to a 96-well microtiter plate. To allow the binding of recombinant immunotoxin to CD64, the recombinant immunotoxin was added to the wells at different concentrations (0, 1, 2, 4, 8 and 10 μg/mL). By washing the wells in between incubations, rabbit anti-HALT-1 primary antibody (2.3 μg/mL), goat anti-rabbit IgG-AP (12.5 ng/mL) and 4-MUP (4-methylumbelliferyl phosphate) (Sigma-Aldrich, USA) were added to the wells in a precise sequence of steps. The fluorescence absorbance/exciting readings at 355/460 nm were measured by a multi-mode microplate reader (BMG Labtech, Germany) at 5 min time-point for 30 min. The same experiment was performed without CD64 to ensure that any fluorescence detection is due to the specific binding between CD64 and recombinant immunotoxin. Three negative controls (1) CD64 coated only (2) without primary antibody and (3) without secondary antibody were also included in each set of tests.

### Statistical data analysis

ELISA of HALT-1-scFv was conducted in biological and technical triplicates for each CD64-coated and CD64-uncoated wells. The statistical analysis was performed by using R version 3.6.0 [47] (https://www.R-project.org/). The consistency of biological triplicate experiments was examined by using One-Way Repeated Measures ANOVA. In order to distinguish the specificity of HALT-1-scFv to CD64-coated wells from uncoated wells, one tail paired T test was conducted. For all the test conducted, *p* ≤ 0.05 was set to define significance of differences.

### Polarization of M1-like macrophages

Cytotoxicity assay of the recombinant immunotoxins was performed on CD64^+^ THP-1 (ATCC TIB-202) human monocyte cell line. Cells were routinely cultured in 20 mL of Roswell Park Memorial Institute (RPMI 1640) culture medium (Nacalai tesque, Japan) supplemented with 10% fetal bovine serum (FBS), 100 U/ml penicillin/streptomycin, 10 mM hepes, 1 mM pyruvate, and 50 pM 2-mercaptoethanol; and grown at 37 °C with 5% CO_2_. For cytotoxicity assay, cells were seeded at 1 × 10^4^ cells/well in 96-well microtiter plate with the addition of 200 nM phorbol 12-myristate 13-acetate (PMA) and incubated for 72 h to activate the monocytes to macrophages. Activated cells were then polarized to M1-like macrophages by changing the medium to fresh RPMI medium containing 20 ng/mL of IFN-γ and 10 pg/mL of LPS, followed by incubation for another 48 h. Expression of CD64 in M1-like polarized macrophages were validated by PCR. CD64 specific primers [48] were used to determine the expression of CD64 and GAPDH specific primers [49] were used for amplifying the positive control.

### In vitro cytotoxicity assay

CD64^+^ M1-like macrophages were treated with various concentrations of recombinant immunotoxin (5, 10, 15, 20, 25 and 30 μg/mL). Three controls (1) medium only (negative), (2) medium with cells (negative) and (3) cells added with dimethyl sulfoxide (DMSO) (positive) were included in each set of assays. CD64^−^ HeLa cells were also treated with the same serial concentration of recombinant immunotoxin to assess the unspecific cytotoxicity. MTT (3-(4,5-dimethylthiazol-2-yl)-2,5-diphenyltetrazolium bromide) stock solution (5 mg/mL) was added to each well and incubated for another 3 h at 37 °C with 5% CO_2_. The formazan violet crystals were dissolved by addition of 200 μL DMSO (99.5%) followed by measurement at 570 nm with the reference of 630 nm using a spectrophotometric microplate reader (Bio-Tek, USA).

## Supplementary information

**Additional file 1: Supplementary Table 1.** List of OE-PCR primers. **Supplementary Table 2.** Refolding buffers components. **Supplementary Table 3.** Yield of recombinant immunotoxins before and after refolding

**Additional file 2: Figure S1.** 12% SDS-PAGE image and binding assay of a-CD64 scFv. These are the original gel images shown in Fig. 1a and b. a Expression of recombinant a-CD64-scFv. Lane 1, 10–250 kDa protein ladder; lane 2, soluble fraction; lane 3, insoluble fraction. The expected band of 32 kDa was observed in the insoluble fraction. b a-CD64-scFv after refolding in a series of deceasing urea concentrations. Lane 1, protein ladder, lane 2, *E. coli* cell lysate with the induction of IPTG; lane 3, *E. coli* cell lysate without IPTG, and lane 4, refolded a-CD64 scFv visible as the band of 32 kDa. **Figure S2.** 12% SDS-PAGE of the recombinant immunotoxins showing their expression, solubility and refolding yield. These images are the original gel images shown in Fig. 3. Lanes that are not labelled have no direct relevance to the data presented in this study. a Cell lysate was extracted after the expression of recombinant scFv-HALT-1 in BL21(DE3) *E. coli* cells. Lane 1, 10–250 kDa protein ladder; lane 2, scFv-HALT-1 in the presence of IPTG; lane 3, scFv-HALT-1 in the absence of IPTG.b Cell lysate was extracted after the expression of recombinant HALT-1-scFv in BL21(DE3) *E. coli* cells. Lane 1, 10–250 kDa protein ladder; lane 2, HALT-1-scFv in the presence of IPTG; lane 3, HALT-1-scFv in the absence of IPTG. c Solubility of HALT-1-scFv was examined after the cell disruption by sonication. Lane 1, 10–250 kDa protein ladder; lane 2, HALT-1-scFv insoluble faction; lane 3, HALT-1-scFv soluble fraction. d Solubility of scFv-HALT-1 was examined after the cell disruption by sonication. Lane 1, 10–250 kDa protein ladder; lane 2, scFv-HALT-1 insoluble faction; lane 3, scFv-HALT-1 soluble fraction. e Recombinant HALT-1-scFv after the refolding process. Lane 1, 12–120 kDa protein ladder; lane 2, HALT-1-scFv. f Recombinant scFv-HALT-1 after the refolding process. Lane 1, 12–120 kDa protein ladder; lane 2, scFv-HALT-1. **Figure S3.** PCR validation of CD64 expression. Gel electrophoresis images are not the original image of Fig. 5a but they were derived from two repeated experiments as that of Fig. 5a. For both a and b, lane 1, 1 kb plus DNA ladder; lane 2, CD64 expression in M1-like macrophage; lane 3, CD64 expression in HeLa cells; lane 4, GAPDH expression in M1-like macrophage; lane 5, GAPDH expression in HeLa cells.

## Data Availability

The datasets used and/or analysed during the current study are available from the corresponding author on reasonable request.

## Abbreviations

4-MUP: 4-methylumbelliferyl phosphate
α-PFT: α-pore forming toxin
ANOVA: Analysis of variance
BSA: Bovine serum albumin
CD64: Cluster of differentiation 64
DMSO: Dimethyl sulfoxide
ELISA: Enzyme-linked immunosorbent assay
ETA: Exfoliative toxin A
FBS: Fetal bovine serum
GAPDH: Glyceraldehyde 3-phosphate dehydrogenase
HALT-1: Hydra actinoporin-like toxin 1
HRP: Horseradish peroxidase
IC50: Half maximal inhibitory concentration
IFN-γ: Interferon gamma
IgG-AP: Immunoglobulin G conjugated with alkaline phosphatase
IPTG: Isopropyl β-D-1-thiogalactopyranoside
kDa: Kilo dalton
MAP: Microtubule-associated protein tau
MTT: 3-(4,5-dimethylthiazol-2-yl)-2,5-diphenyltetrazolium bromide
OE-PCR: Overlap extension polymerase chain reaction
PBS: Phosphate buffered saline
PMA: Phorbol 12-myristate 13-acetate
PMSF: Phenylmethylsulfonyl fluoride
RPMI: Roswell Park Memorial Institute
scFv: Single-chain variable fragment
SDS-PAGE: Sodium dodecyl sulfate polyacrylamide gel electrophoresis
TMB: 3,3′,5,5′-tetramethylbenzidine
