# Supplementary material for: Evaluation of Hydra HALT-1 as a toxin moiety for recombinant immunotoxin
Source: BMC Biotechnol. 2020 Jun 17;20:31. doi: 10.1186/s12896-020-00628-9 (PMC7301450; doi:10.1186/s12896-020-00628-9)
Supplement: Supplementary file 1 — Additional file 1: Supplementary Table 1. List of OE-PCR primers. Supplementary Table 2. Refolding buffers components. Supplementary Table 3. Yield of recombinant immunotoxins before and after refolding [file 12896_2020_628_MOESM1_ESM.docx]

**The construction of immunotoxins scFv-HALT-1 and HALT-1-scFv: sequence information for primers used in the overlap extension polymerase chain reaction**

**Supplementary Table 1** List of OE-PCR primers

| Primer name | Sequence^a^ |
| --- | --- |
| *N-terminal HALT-1 and C-terminal scFv* | |
| HALT-1 Fw (*Nco*I) | 5’GAATAAGCCATGGCTCATCATCATCATCATCATCTGGTGCCGCGCGGCAGCGCAGCTTTAGGAGTTATAGC’3 |
| HALT-1 Rv (linker) | 5’**AGAACCACCACCTCCAGAACCACCACCACCAGATCCACCACCACCAGA**TCCAGAAAAAATAACTTTGA ‘3 |
| α-CD64-scFv Fw (linker) | 5’**TCTGGTGGTGGTGGATCTGGTGGTGGTGGTTCTGGAGGTGGTGGTTCT**ATGGCGCAAGTGCAGTTGGT ‘3 |
| α-CD64-scFv Rv (*Xho*I) | 5’GTTGCCCTCGAG*TCA*TTTGATTTCGAGTTTGGTGC’3 |
| *N-terminal scFv and C-terminal HALT-1* | |
| α-CD64-scFv Fw (*Nco*I) | 5’GAATAAGCCATGGCTCATCATCATCATCATCATCTGGTGCCGCGCGGCAGCATGGCGCAAGTGCAGTTGGT’3 |
| α-CD64-scFv Rv (linker) | 5’**AGAACCACCACCTCCAGAACCACCACCACCAGATCCACCACCACCAGA**TTTGATTTCGAGTTTGGTGC ‘3 |
| HALT-1 Fw (linker) | 5’**TCTGGTGGTGGTGGATCTGGTGGTGGTGGTTCTGGAGGTGGTGGTTCTGCAGCTTTAG**GAGTTATAGC ‘3 |
| HALT-1 Rv (*Xho*I) | 5’GTTGCCCTCGAGTCATCCAGAAAAAATAAC  TTTGA’3 |

^a^ Underlined sequence refers to restriction enzyme site. Sequence coding for six histidine residues are squared in a box. Bold underlined sequence refers to the linker sequence.

**Optimised buffers in the stepwise dialysis for refolding of recombinant immunotoxins**

**Supplementary Table 2** Refolding buffers components

| Urea (M) | Sodium Phosphate  (mM) | NaCl  (M) | L-arginine  (M) | 2-mercaptoethanol  (mM) |
| --- | --- | --- | --- | --- |
| 6.0 | 20 | 0.5 | 0.1 | 50 |
| 4.0 | 20 | 0.5 | 0.1 | 50 |
| 2.0 | 20 | 0.5 | 0.5 | 50 |
| 1.0 | 20 | 0.5 | 0.5 | 50 |

**A comparison of recombinant immunotoxin yields before and after refolding procedure and the recovery efficiency of immunotoxin from refolding**

**Supplementary Table 3** Yield of recombinant immunotoxins before and after refolding

| Recombinant immunotoxin | Total yield after purification (µg) | Total yield after refolding (µg) | Refolding efficiency (%) |
| --- | --- | --- | --- |
| HALT-1-scFv | 2115 | 766.7 | 36.2 |
| scFv-HALT-1 | 1557.5 | 390 | 25 |
