# Supplementary material for: Evaluation of Hydra HALT-1 as a toxin moiety for recombinant immunotoxin
Source: BMC Biotechnol. 2020 Jun 17;20:31. doi: 10.1186/s12896-020-00628-9 (PMC7301450; doi:10.1186/s12896-020-00628-9)
Supplement: Supplementary file 2 — Additional file 2: Figure S1. 12% SDS-PAGE image and binding assay of a-CD64 scFv. These are the original gel images shown in Fig. 1a and b. a Expression of recombinant a-CD64-scFv. Lane 1, 10–250 kDa protein ladder; lane 2, soluble fraction; lane 3, insoluble fraction. The expected band of 32 kDa was observed in the insoluble fraction. b a-CD64-scFv after refolding in a series of deceasing urea concentrations. Lane 1, protein ladder, lane 2, E. coli cell lysate with the induction of IPTG; lane 3, E. coli cell lysate without IPTG, and lane 4, refolded a-CD64 scFv visible as the band of 32 kDa. Figure S2. 12% SDS-PAGE of the recombinant immunotoxins showing their expression, solubility and refolding yield. These images are the original gel images shown in Fig. 3. Lanes that are not labelled have no direct relevance to the data presented in this study. a Cell lysate was extracted after the expression of recombinant scFv-HALT-1 in BL21(DE3) E. coli cells. Lane 1, 10–250 kDa protein ladder; lane 2, scFv-HALT-1 in the presence of IPTG; lane 3, scFv-HALT-1 in the absence of IPTG.b Cell lysate was extracted after the expression of recombinant HALT-1-scFv in BL21(DE3) E. coli cells. Lane 1, 10–250 kDa protein ladder; lane 2, HALT-1-scFv in the presence of IPTG; lane 3, HALT-1-scFv in the absence of IPTG. c Solubility of HALT-1-scFv was examined after the cell disruption by sonication. Lane 1, 10–250 kDa protein ladder; lane 2, HALT-1-scFv insoluble faction; lane 3, HALT-1-scFv soluble fraction. d Solubility of scFv-HALT-1 was examined after the cell disruption by sonication. Lane 1, 10–250 kDa protein ladder; lane 2, scFv-HALT-1 insoluble faction; lane 3, scFv-HALT-1 soluble fraction. e Recombinant HALT-1-scFv after the refolding process. Lane 1, 12–120 kDa protein ladder; lane 2, HALT-1-scFv. f Recombinant scFv-HALT-1 after the refolding process. Lane 1, 12–120 kDa protein ladder; lane 2, scFv-HALT-1. Figure S3. PCR validation of CD64 expression. Gel electrophoresis images [file 12896_2020_628_MOESM2_ESM.pdf]

a

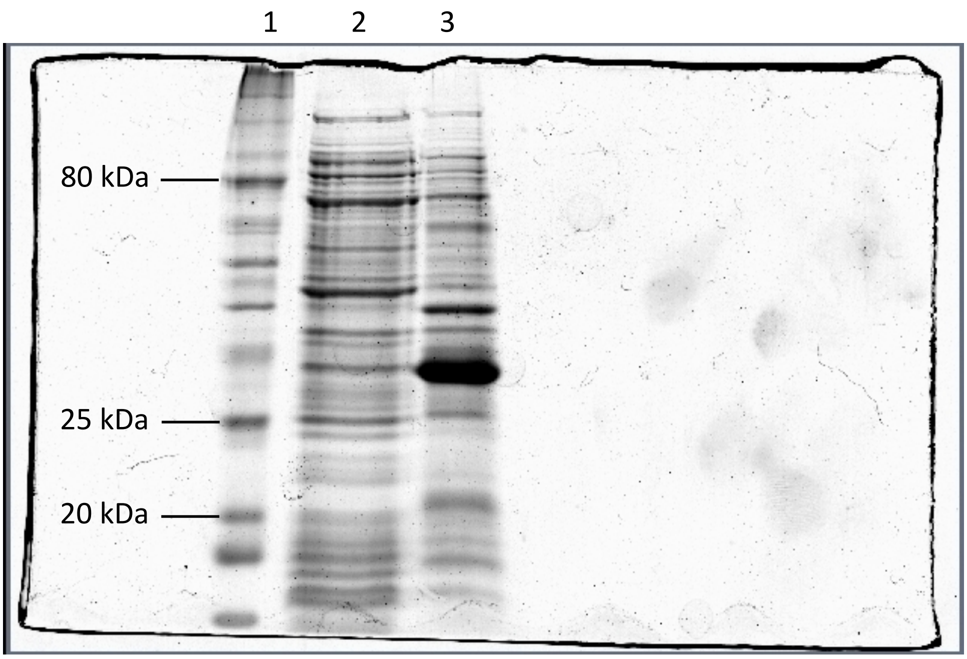

b

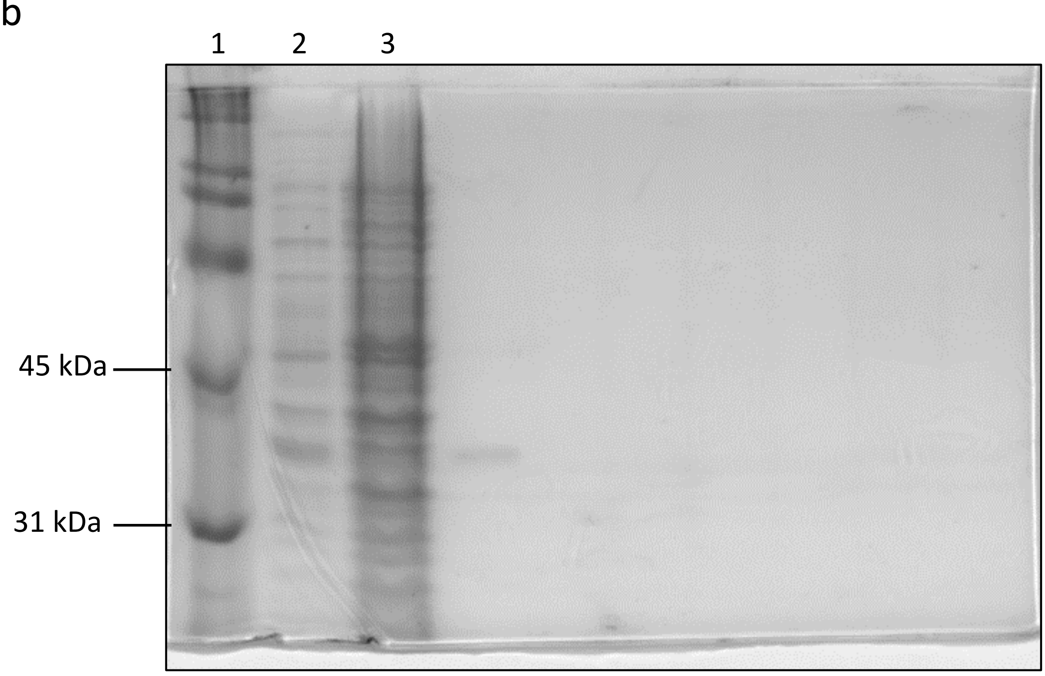

Figure S1 12% SDS-PAGE image and binding assay of α-CD64 scFv. These are the original gel images shown in Fig. 2a and b. **a** Expression of recombinant α-CD64-scFv. Lane 1, 10-250 kDa protein ladder; lane 2, soluble fraction; lane 3, insoluble fraction. The expected band of 32 kDa was observed in the insoluble fraction. **b** α-CD64-scFv after refolding in a series of deceasing urea concentrations. Lane 1, protein ladder, lane 2, *E. coli* cell lysate with the induction of IPTG; lane 3, *E. coli* cell lysate without IPTG, and lane 4, refolded α-CD64 scFv visible as the band of 32 kDa.

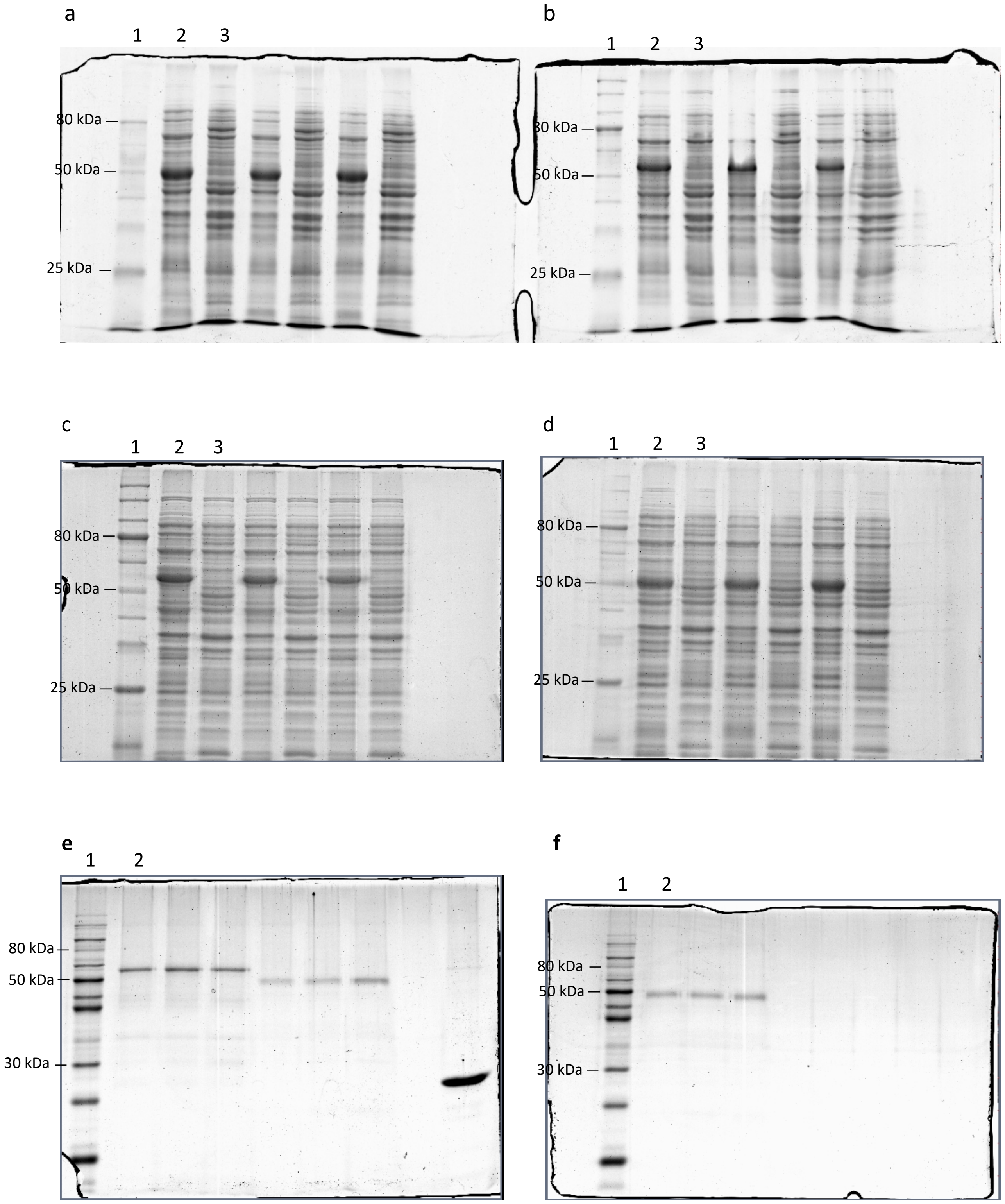

Figure S2 12% SDS-PAGE of the recombinant immunotoxins showing their expression, solubility and refolding yield. These images are the original gel images shown in figure 3. Lanes that are not labelled have no direct relevance to the data presented in this study. **a** Cell lysate was extracted after the expression of recombinant scFv-HALT-1 in BL21(DE3) *E. coli* cells. Lane 1, 10-250 kDa protein ladder; lane 2, scFv-HALT-1 in the presence of IPTG; lane 3, scFv-HALT-1 in the absence of IPTG.

b Cell lysate was extracted after the expression of recombinant HALT-1-scFv in BL21(DE3) *E. coli* cells. Lane 1, 10-250 kDa protein ladder; lane 2, HALT-1-scFv in the presence of IPTG; lane 3, HALT-1-scFv in the absence of IPTG. **c** Solubility of HALT-1-scFv was examined after the cell disruption by sonication. Lane 1, 10-250 kDa protein ladder; lane 2, HALT-1-scFv insoluble faction; lane 3, HALT-1-scFv soluble fraction. **d** Solubility of scFv-HALT-1 was examined after the cell disruption by sonication. Lane 1, 10-250 kDa protein ladder; lane 2, scFv-HALT-1 insoluble faction; lane 3, scFv-HALT-1 soluble fraction. **e** Recombinant HALT-1-scFv after the refolding process. Lane 1, 12-120 kDa protein ladder; lane 2, HALT-1-scFv. **f** Recombinant scFv-HALT-1 after the refolding process. Lane 1, 12-120 kDa protein ladder; lane 2, scFv-HALT-1.

a

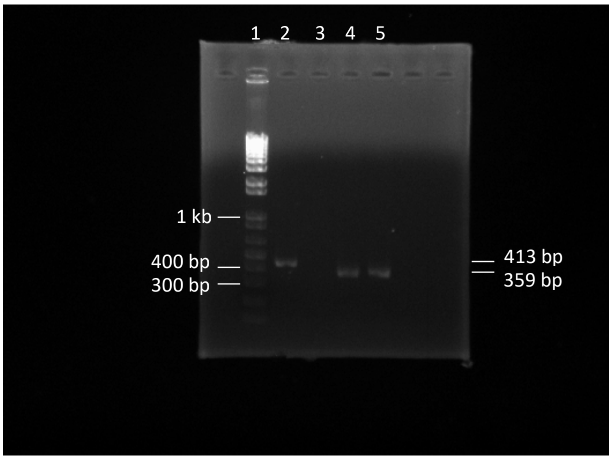

b

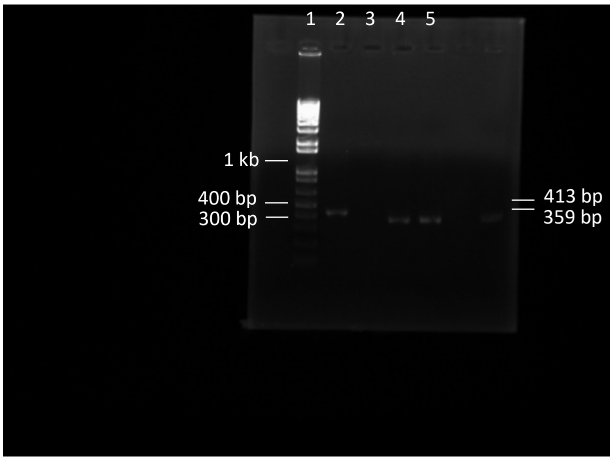

Figure S3 PCR validation of CD64 expression. Gel electrophoresis images are not the original image of Fig. 5a but they were derived from two repeated experiments as that of Fig. 5a. For both **a** and **b**, lane 1, 1kb plus DNA ladder; lane 2, CD64 expression in M1-like macrophage; lane 3, CD64 expression in HeLa cells; lane 4, GAPDH expression in M1-like macrophage; lane 5, GAPDH expression in HeLa cells.
